# 

**Published:** 1902-12-27

**Authors:** 


### NOTICE TO CORRESPONDENTS.

All MSS., Letters, Books for Review, and other matters intended for the Editor should be addressed The Editor, "The Hospital," The Hospital Building, 28 & 29 Southampton Street, Strand, W.C.

The Editor cannot undertake to return rejected MS., even when accompanied by stamped directed envelope.

**Notice to Advertisers and Subscribers.**

All Advertisements, Orders for Copies of The Hospital, and Business Communications should be addressed to **THE MANAGER, (Not to the Editor)**, The Hospital, 28 & 29 Southampton Street, Strand, London, W.C.

The Cost of Subscription, post free, is as follows.—Per week, 2½d.; per quarter, 2s. 8d.; per half-year, 5s. 3d.; per annum, 10s. 6d. Foreign:—Per annum, 15s. 2d., payable, in all cases, in advance.

**NOTICE.—Vol. XXXII. of "The Hospital" (April to September 1902) in handsome cloth binding is now on sale at the office of this Journal, price, 7s. 6d. Cases for binding the Numbers contained in this Volume 1s. 6d. Index to Vol. XXXII., 2½d., post free.**

**The Monthly Part for January will be ready on January 1. Price 6d., by post 8d.**

### THE STUDENT OF MEDICINE.

### APPOINTMENTS.

**Arthur Anderson**, M.B., Ch.M.Syd., appointed Junior Medical Officer in the Department of Lunacy, New South Wales. **J. Blumfeld**, M.D.Cantab., appointed Anæsthetist to the Hospital for Epilepsy and Paralysis, Maida Vale. **Herbert Child**, M.R.C.S.-Eng., appointed Surgeon to the Department for Diseases of the Throat, Nose, and Ear, Reading Dispensary. **Alexander Y. Fullerton**, F.R.C.S., L.R.C.P.Edin., appointed Government Medical Officer and Vaccinator at Murrurandi, New South Wales. **Geo. G. Hamilton**, M.B., F.R.C.S.Eng.&Edin., appointed Honorary Surgeon to the Liverpool Royal Infirmary. **Wilfrid J. Harris**, M.D.Camb., M.R.C.P.Lond., appointed Physician to Out-patients and Registrar to the Hospital for Epilepsy and Paralysis, Maida Vale. **D. MacMaster**, M.B, Ch.M.Syd., M.R C.S., appointed Honorary Assistant Surgeon to the Sydney Hospital for Sick Children, New South Wales. **D. M. Mackenzie**, M.B., Ch.B.Aberd., appointed Junior Resident Medical Officer of Croydon General Hospital. **Henry H. McWilliams**, L.R C.P., appointed Health Officer for the Shire of Lancefield, Victoria. **John A. O'Brien**, M.B., appointed Acting Medical Superintendent, Kew Hospitals for the Insane, Victoria. **L. Hemington Pegler**, M.D.Edin., M.R.C.S., appointed Honorary Surgeon for Diseases of the Ear, Nose, and Throat to the Clergy Orphan Corporation School for Girls, Bushey, Herts. **Frederick Peipers**, M.D., appointed Health Officer for the Shire of Whittlesea, Victoria. **Hugh R. Phillips**, M.D.Edin., appointed Second Anæsthetist to the Italian Hospital, Queen Square. **Richard Rendle**, F.R.C.S.-Eng., appointed Medical Officer at Cloncurry, Queensland. **J. F. Robinson**, F.R.C.S.Eng., L.R.C.P.Lond., appointed Senior Resident Medical Officer of Croydon General Hospital.

**"The Hospital" Institutional Library.**

"Hospitals and Asylums of the World." 4 Vols., with a Portfolio of Plans, complete £12 12s.

"Burdett's Hospitals and Charities, 1902." 5s.

"Cottage Hospitals, General, Fever, and Convalescent." 10s. 6d.

"Hospital Expenditure: The Commissariat." 2s. 6d.

"A Legal Handbook for the Use of Hospital Authorities." 2s. 6d.

"The Cottage Hospital Case Book or Register of Patients." 10s. and 12s. 6d.

"The Furnishing and Appliances of a Cottage Hospital." 6d.

All these are published by the Scientific Press, Ltd., and may be obtained through any bookseller, or direct from the publishers, 28 and 29 Southampton Street, Strand, London, W.C.

